# Predictors of Developing Significant Mitral Regurgitation Following Percutaneous Mitral Commissurotomy with Inoue Balloon Technique

**DOI:** 10.4061/2011/703515

**Published:** 2011-08-15

**Authors:** Abdelfatah A. Elasfar, Hatem F. Elsokkary

**Affiliations:** ^1^Cardiology Department, Faculty of Medicine, Tanta University, Egypt; ^2^King Fahad Medical City, Riyadh, Saudi Arabia

## Abstract

*Background*. Despite the high technical expertise in percutaneous mitral commissurotomy (PMC), mitral regurgitation (MR) remains a major procedure-related complication. The aim of this work is to find out the most sensitive and applicable predictors of development of significant mitral regurgitation (SMR) following percutaneous mitral commissurotomy using Inoue balloon technique. *Methods*. We studied prospectively the preprocedural (clinical, echocardiography, and hemodynamic) and procedural predictors of significant mitral regurgitation (identified as increase of ≥2/4 grades of pre-PMC MR by color Doppler flow mapping) following valvuloplasty using Inoue balloon in 108 consecutive patients with severe mitral stenosis. Multiple stepwise logistic regression analysis was performed for variables found positive on univariate analysis to determine the most important predictor(s) of developing SMR. *Results*. The incidence of SMR following PMC using Inoue technique was 18.5% (10 patients). MV scoring systems were the only variables that showed significant differences between both groups (Group A without SMR and Group B with SMR). However, no clinical, other echocardiographic measurements, hemodynamic or procedural variables could predict the development of SMR. Using multiple regression analysis, the best predictive factor for the risk of SMR after Inoue BMV was the total MR-echo score with a cutoff point of 7 and a predictive percentage of 97.7%. *Conclusions*. The total MR-echo score is the only independent predictor of SMR following PMC using Inoue technique with a cutoff point of 7.

## 1. Introduction

Rheumatic fever is the most common cause of acquired heart diseases in children, young, and middle-aged adults worldwide. The disease is endemic in developing countries including Middle East countries [1]. Mitral stenosis (MS) is almost invariably the result of chronic rheumatic heart disease secondary to one or more prior episodes of acute rheumatic fever [1]. Since Inoue et al. introduced balloon valvuloplasty in 1984, this procedure has become the treatment of choice replacing surgical commissurotomy in many cases [2]. Despite the high technical expertise in percutaneous mitral commissurotomy (PMC), mitral regurgitation (MR) remains a major procedure-related complication [3]. The incidence of severe MR after PMC in the literature varies between 1.4% and 7.5% [4–6]. Many studies using different techniques of PMC have identified mitral valve anatomy as a predictive factor of the immediate results. However, the global echocardiographic classifications have some limitations [7]. This study aimed at finding out the most sensitive and applicable predictors of the development of significant mitral regurgitation (SMR) following PMC using Inoue balloon technique whether preprocedural or procedural. 

## 2. Methods

We prospectively included 108 consecutive patients with rheumatic mitral stenosis who are subjected to PMC using Inoue balloon technique during the period from February 2007 to October 2009 in two centers in the Middle East, Tanta University Hospital in Egypt and King Fahad Medical City in Saudi Arabia. Our study is conformed to the Helsinki Declaration. The Institutional Review Board (IRB) approved the study in both centers. All patients gave informed consent to participate in the study.

Inclusion criteria included symptomatic MS with NYHA functional class II or more with mitral valve area (MVA) ≤1.5 cm^2^ and mitral valve Echocardiographic score ≤11 according to scoring system described by Wilkins et al. [8]. Exclusion criteria were patients with MR or aortic regurgitation (AR) of grade II/IV or more, also, patients with Wilkins scoring of >11 [8] and presence of left atrial (LA) thrombi assessed by transesophageal echocardiography (TEE). To avoid personal bias in assessing the anatomic scores and the mitral valves, these parameters were graded by at least two readers and in case of disparity by a third one to ensure correct classification. 

Patients were divided into two groups according to the development of SMR or not: *Group A* (patients without SMR) and *Group B* (patients who developed SMR). All patients were subjected to full history taking and clinical examination, standard resting 12-lead electrocardiogram.

Transthoracic echocardiographic and Doppler examinations were done within a week before intervention and one day after the procedure. Examination was acquired with the patients in the left lateral decubitus position by using a commercially available system (Vingmed Vivid Seven, General Electric—Vingmed, Milwaukee, WI, USA) equipped with 2.5 and 3.5 MHz transducers and 5 MHz transesophageal monoplane transducer. 

We studied mitral valve morphology using different scoring systems including Massachusetts General Hospital score by Wilkins et al. [8], MR-echo score by Padial et al. [9], and Commissural score by Sutaria et al. [10]. We also measured mitral valve area (MVA), LA dimensions, transmitral maximum pressure gradient (MG), estimated systolic pulmonary artery pressure (SPAP), and presence of MR and its severity. Severity of MR was determined by expressing the ratio of maximal jet area to left atrial area in the same view using color flow mapping and graded from one to four according to Essop et al. [11]. SMR was defined as the increase of ≥2/4 grades of MR. Furthermore, other quantitative measures such as vena contracta or PISA methods were used to assess the severity of MR in cases of uncertainty about the use of maximal regurgitant jet area.

Transesophageal echocardiography (TEE) was done one day preintervention for exclusion of LA and LA appendage thrombi, measuring of interatrial septal thickening, and reassessment of transthoracic echocardiographic data.

Invasive hemodynamic study included predilatation and early postdilatation assessment of LA pressure, left ventricular end-diastolic (LVED) pressure, and transmitral pressure gradient (PG). 

PMC using Inoue technique was done as described before in the literature [12].

## 3. Statistical Analysis

Data are presented as mean ± SD. Categorial variables were compared using Student's *t-*test. Nonparametric data were compared using Chi-square test. Multiple stepwise logistic regression analysis was performed for variables found positive on univariate analysis to determine the most important predictor(s) of developing SMR. *P* value was considered significant when it was <0.05. Data were collected using SPSS version 16.

## 4. Results

This study prospectively included consecutive 108 patients with mitral stenosis who were subjected to PMC using Inoue technique. Using Doppler color flow imaging before the procedure, 94 patients (87%) had no MR and 14 patients had (13%) grade +1 MR. After the procedure, 36 patients (33.3%) still had no MR and 52 patients (48.2%) had grade +1 MR. However, 2 patient (1.8%) developed grade +2 MR, 8 patients (7.4%) grade +3 MR, and 10 patients (9.3%) grade +4 MR. So, the incidence of postprocedure SMR and severe MR in our study was 18.5% and 9.3%, respectively. According to the development of SMR, we divided our patients into two groups (*Group A*, 88 patients without SMR and *Group B*, and 20 patients with SMR). There were no significant differences between both groups regarding their baseline clinical characteristics, echocardiographic measurements other than MV scoring, preprocedural hemodynamic, and procedural data (Tables 1 and 2).

MV Morphology and Scoring Systems (Table 3):

- *Wilkins Score*: the calcification score and the total score were significantly higher in group B than in Group A (*P* = 0.006, 0.028, resp.);
- *MR-echo Score*: the total score and all of its components except subvalvular disease were significantly higher in Group B (*P* = 0.001);
- *Commissural Score*: it was also significantly higher in group B (*P* = 0.003).

### 4.1. Predictors of SMR

As the MV scoring systems were the only variables that showed significant differences between both groups, and using multiple regression analysis, we found that the best predictive factor for the risk of SMR after Inoue BMV is the total MR-echo score with a cutoff point of 7 and a predictive percentage of 97.7%. 

## 5. Discussion

The large number of patients with mitral stenosis (MS) may require some modality of invasive treatment during their life [13]. The therapeutic approach to MS has evolved considerably since 1984, after the first report of percutaneous balloon mitral valvuloplasty by Inoue et al. [2]. Since that time, percutaneous balloon mitral valvuloplasty has become a common treatment of mitral stenosis [2]. Severe MR after PMC remains one of the most important complications of this procedure [6]. Risk of SMR is defined as an increase of ≥2/4 grade of MR [11]. However, the precise factors predicting SMR are not fully understood and rather controversial [11]. 

The present study prospectively included 108 patients with rheumatic mitral stenosis who were subjected to PMC using Inoue balloon technique. They were divided according to the development of SMR into two groups, *Group A* (88 patients without SMR) and *Group B* (20 patients with SMR).

In our study, the incidence of SMR was 18.5% and that of severe MR was 9.3% following PMC with Inoue technique. Echo-Doppler clarified the mechanisms of development of SMR as follow: a tear of the posterior mitral leaflet in 10 patients, a tear of the anterior leaflet in 8 patients, and 1 patient had chordal rupture. The incidence of severe MR after balloon mitral valvotomy in the literature varies between 1.4% and 7.5% [4–6]. Zaki et al. reported postprocedural severe MR in 9.8% of patients with MS undergone balloon mitral valvuloplasty (BMV) [14]. Similarly, Kasem et al. reported that the incidence of severe MR after PMC using Inoue balloon technique was 10% [15]. Hernandez et al. reported that 31% of their patients had an increase in MR grade but only 6.6% developed severe MR [4]. Also, Mezillis et al. reported severe MR in 9% of their patients [16]. So, our results regarding the incidence of severe MR are comparable to most of previously done studies. Regarding the incidence of SMR and in agreement with our results (18.5%), that of Essop et al. was 17.5% and Mezillis et al. was 17% of their patients [11, 16].

There were no significant differences between both groups regarding baseline clinical data, echocardiographic measurements other than MV scoring, preprocedural hemodynamic data, or procedural (technical) data. These results came in agreement with several previous studies where no clinical, echocardiographic (with exception of MV Scoring), procedural, or hemodynamic predictors for the development of SMR or severe MR following Inoue BMV were found [4, 5, 11, 17–19]. In contradictory to the results of the present study, smaller MVA, higher MG and LA dimension were proposed by Mailer et al. to be predictors for the development of MR following Inoue BMV [20]. Also, Cho et al. reported the initial MVA as a determinant of increase MR following Inoue technique [21]. 

Regarding Wilkins score, both the calcification score and total score were significantly higher in group B (those developed SMR). For MR-echo score, all the anterior leaflet, posterior leaflet, and total scores were higher in group B. Also, the commissural score was significantly higher in group B. So, using backward elimination logistic regression analysis, we applied three predictive models for SMR after PMC with Inoue technique. From these three models, the best predictive factor for the risk of SMR was the total MR-echo score. In agreement with our results, several previous trials failed to predict SMR following PMC with Inoue technique using total Wilkins score [11, 19, 22–25].

 However, in contradictory to our results, Chen et al. identified the severity of subvalvular disease as an independent predictor of increase MR severity after BMV [26]. García-Castillo et al. identified total Wilkins score >8 as an independent predictor of severe MR which is also against our results [27]. However, both García-Castillo et al. [27] and Inoue et al. [28] have some agreement with our results where commissural calcification was a predictor of developing SMR. Regarding commissural score, the results of Sutaria et al. [10] came in agreement with our results that failed to use it as a predictor of development of SMR following BMV with Inoue technique. 

Regarding MR-echo score, Padial et al. identified it as the only independent predictor for developing severe MR after Inoue technique using a total score of >10 as a cutoff point [9]. Also, Mezilis et al. reported that MR-echo score is better at reliably identifying patients at risk of developing SMR after the Inoue technique with sensitivity and specificity of 83% and 100%, respectively, using 8 as a cutoff point [16]. These results came in agreement with our study, although the cutoff point in our study was 7. Limitations: we tested SMR following PMC using only Inoue technique but not other techniques such as multitrack balloon technique. Also, we did not evaluate other new MV scoring systems such as Cormier's grading of mitral valve anatomy [29] and that developed by Rifaie et al. [30].

## 6. Conclusions

We concluded from our study that no baseline clinical characteristics, preprocedural hemodynamic data, or procedural techniques were predictors of SMR following Inoue BMV. Regarding echocardiographic predictors of SMR, the total MR-echo score developed by Padial et al. is the only independent predictor of SMR following PMC using Inoue technique with a cutoff point of 7.

## Figures and Tables

**Table 1:** Baseline Characteristics.

Variable	Group A (*n* = 88)	Group B (*n* = 20)	*P* value
Clinical Characteristics:			
(i) Age (Years)	31 ± 9	32 ± 10	0.86
(ii) Male/Females	22/66	6/14	0.58
(iii) Height	159 ± 7	157 ± 5	0.81
(iv) BSA	1.6 ± 0.2	1.6 ± 0.3	0.92
(v) Prior PMC	13.6%	15.8%	0.95
(vi) NYHA Class	2.6 ± 0.7	2.7 ± 0.8	0.94
(vii) AF	27.2%	31.6%	0.59

Pre-PMC Echo-Doppler data (except MV Scoring):			
(i) MVA (cm^2^)	0.9 ± 0.2	0.8 ± 0.2	0.37
(ii) MG	14 ± 3	13 ± 2	0.71
(iii) SPAP	44 ± 20	41 ± 21	0.67
(iv) LA dimension	4.8 ± 1.0	4.9 ± 0.7	0.47

BSA: body surface area; NYHA: New York Heart Association classification; AF: atrial fibrillation. *Significant at <0.05

**Table 2:** Hemodynamic and procedural data.

Variable	Group A (*n* = 88)	Group B (*n* = 20)	*P *value
Pre-Procedural Hemodynamic Data:			
(i) LAP	25.8 ± 6.6	30.3 ± 5.7	0.16
(ii) LVEDP	6.3 ± 2.0	5.9 ± 2.4	0.57
(iii) Transmitral PG	20.5 ± 6.8	24.3 ± 8.1	0.14

Procedural (Technical) Data:			
(i) Balloon Size	26.5 ± 0.9	26.0 ± 1.1	0.27
(ii) No. of Inflations	2.0 ± 0.9	2.1 ± 1.1	0.28

*Significant at <0.05

**Table 3:** MV Scoring Systems.

Variable	Group A (*n* = 88)	Group B (*n* = 20)	*P* value
(A) Wilkins Score:			
(i) Mobility	2.4 ± 0.5	2.7 ± 0.9	0.38
(ii) Thickening	2.2 ± 0.4	2.3 ± 0.5	0.59
(iii) Subvalvular	1.5 ± 0.5	1.7 ± 0.7	0.48
(iv) Calcification	0.9 ± 0.7	2.0 ± 0.7	0.006*
(v) Total Score	7.1 ± 1.7	8.9 ± 2.1	0.028*

(B) MR-echo Score:			
(i) Anterior Leaflet	1.8 ± 0.7	2.5 ± 0.5	0.024*
(ii) Posterior Leaflet	1.6 ± 0.6	2.5 ± 0.7	0.010*
(iii) Commissures	1.2 ± 0.5	2.5 ± 1.1	0.002*
(iv) Subvalvular	1.5 ± 0.5	1.7 ± 0.7	0.49
(v) Total Score	6.2 ± 1.0	9.3 ± 1.4	0.001*

(C) Commissural Score:			
(i) Total Score	0.7 ± 0.6	2.0 ± 0.9	0.003*

*Significant at <0.05

## Abbreviations

PMC: Percutaneous mitral Commissurotomy
MR: Mitral regurgitation
SMR: Significant mitral regurgitation
MS: Mitral stenosis
MVA: Mitral valve area
NYHA: New York Heart Association
TEE: Transesophageal echocardiography
LA: Left atrial
MVA: Mitral valve area.
